# Assessment of theory of mind in Tunisian verbal children with autism spectrum disorder

**DOI:** 10.3389/fpsyt.2022.922873

**Published:** 2022-11-17

**Authors:** Selima Jelili, Soumeyya Halayem, Olfa Rajhi, Zeineb Abbes, Hajer Ben Mansour, Sami Ouanes, Amal Taamallah, Selima Ennaifer, Houda Ben Yahia, Melek Ghazzei, Ahmed Nabli, Malek Hajri, Radhouane Fakhfakh, Ali Mrabet, Asma Bouden

**Affiliations:** ^1^Department of Child and Adolescent Psychiatry, Razi Hospital, Manouba, Tunisia; ^2^Faculty of Medicine, Tunis El Manar University, Tunis, Tunisia; ^3^Department of Child and Adolescent Psychiatry, Versaille Hospital Center, Le Chesnay, France; ^4^Department of Psychiatry - Hamad Medical Corporation, Doha, Qatar; ^5^Department of Epidemiology and Statistics, Abderrahmen Mami Hospital, Tunis, Tunisia; ^6^Department of Epidemiology and Public Health, Tunis, Tunisia

**Keywords:** autism, social cognition, Theory of Mind, children, verbal

## Abstract

The present study examined performance in Theory of Mind (ToM) in a group of 31 Arabic-speaking verbal children (7–12 years-old) with autism spectrum disorder (ASD), in comparison with neurotypical controls (NT) matched for age and for cognitive abilities. An innovative task in a digital format named “The Tunisian Social Situations Instrument” (TSSI) was used and allowed us to study four different subdomains of ToM: attribution of intention and epistemic ToM (cognitive ToM), affective ToM, and detection of faux pas (advanced ToM). Our study showed impairments in ToM in children with ASD, similar to those reported in the literature. Our findings additionally suggested that affective and advanced ToM, specifically the detection of faux pas, might be more challenging for ASD children than other components of ToM. Future studies with larger number of children may lead us to specify which subdomains are the most impaired in order to develop specific tools targeting these specific impairments.

## Introduction

In recent years, a multitude of models have tried to elicit the possible mechanisms underlying cognitive processes in ASD (1). One of these models is the Theory of Mind (ToM). ToM-related hypotheses have led to multiple studies over the last decades in which several subdomains of ToM have been assessed in individuals with ASD. These subdomains included epistemic ToM (false beliefs), attribution of intention, and affective ToM. Epistemic ToM involves the attribution of other people's different (even false) beliefs (2). Attribution of intention helps us understand and interpret other people's behavior or predict their next action (3). Affective ToM consists of understanding others' emotions (4, 5). Several studies assessing all these subdomains reported altered performance in individuals with ASD (2–10). However, the deficit in all these subdomains did not seem to be universal and have led researchers to develop more “subtle” and “advanced” ToM tasks (11).

“Advanced” ToM includes multiple abilities such as recognizing transgressions of social norms, reasoning about ambiguity, bouble bluffs, sarcasm, white lies, and social clumsiness or detection of “faux pas” (12–16).

Social clumsiness consists of a situation occurring when a person behaves inappropriately, or when a speaker says something without considering if it is something that the listener might not want to hear or know. This situation typically has a negative outcome on the listener that the speaker never intended (17). The most used task in the assessment of this subdomain is the Faux-pas test, designed by Baron-Cohen. The task consists of 10 verbally presented faux pas stories describing interpersonal interactions in everyday life situations (17). Using these type of stories, Baron-Cohen et al. (17) showed that children with Asperger Syndrome or high-functioning autism tended to under detect the faux pas. Other studies using the same test reported that adolescents and adults with ASD were able to identify the stories in which a faux pas was committed, suggesting that their ability to reason about others' mental states was relatively preserved. Nevertheless, they failed to provide appropriate justifications of the protagonist's behavior since they never referred to the victim's distress or emotional reaction. According to the authors, these findings can be explained by their inability to integrate the information about the speaker's knowledge state with that concerning the emotional state of the listener (18, 19). These findings were not in accordance with those of 19 suggesting a primary problem with faux pas detection in adults with ASD. Recent papers are still trying to elucidate these mixed results. It is possible that the inconsistency is due to methods used (12, 20) and to differences in verbal abilities, playing an important role in ToM-related performance (9, 21). Furthermore, a recent study has also shown that working memory had a big impact on faux pas understanding in individuals with ASD (22).

The main aim of our research was to contribute to the ongoing research of different aspects of ToM, through: (1) comparing ASD children performance in ToM tasks to matched neurotypical controls, (2) identifying which subdomains are the most altered in ASD, and (3) examining whether performance in ToM tasks is related to verbal abilities. We chose to study ToM in children in particular because the majority of previously published studies targeted adolescents and adults (23–25). In addition, it has been well established that the ability to understand another person's beliefs and reactions (first order ToM) emerges approximately at the age of four to five. Hence, 6- and 7-year-old children are usually able to mentally represent and understand second-order beliefs. In addition, research about the developmental aspects of advanced ToM is still scarce and inconclusive (26).

The effects of language abilities and age on autism have also been well established in the literature [e.g., Van Wijngaarden-Cremers et al. (27)], as having an impact on performance of individuals with ASD [e.g., Georgiades et al. (28), Fletcher-Watson and Happé (29), Lord et al. (30)]. In an effort to control these factors, these variables were used as matching variables in our study.

To our knowledge, this is the first study aiming to examine this cognitive function among Arabic-speaking verbal children (aged 7–12 years) with ASD, comparing them to neurotypical controls (matched for age and for cognitive abilities), using an innovative and recently validated task: “The Tunisian Social Situations Instrument” (TSSI) (31). It seems crucial to include research populations from diverse linguistic backgrounds, in order to maximize the possibilities of generalizing the conclusions of previous research. Furthermore, our study is the first to use one task in digital form to assess 4 different subdomains of ToM including an assessment of 3 aspects of ToM: cognitive, affective, and advanced ToM (detection of faux pas) (31). Studying ToM in children with ASD seems crucial for the construction of remediation tools aiming to improve this function and its different subdomains.

## Methods

### Participants

Our study was conducted between January 2018 and December 2020.

A total of 62 children took part in this study. Thirty-one (32) of these children had ASD and 31 had neurotypical development (NT). All participants were of Tunisian origin, native Arabic speakers, and attended ordinary schools. Groups were matched for age and mental age. For each case (child with ASD), a neurotypical child with the same gender, age, and mental age was randomly selected out of the list of patients who were assessed at the Department of Child and Adolescent Psychiatry - Razi Hospital and who fulfilled the inclusion/exclusion criteria for controls. We did compare groups in terms of all potential covariates that we did not match for (Table 1). Propensity score matching was conducted in the form of ANCOVA and MANCOVA analyses allowing to control for all potential covariates beyond those the groups were initially matched for.

**Table 1:** General characteristics of the autism spectrum disorder group vs. the neurotypical group.

	**ASD group** ***n* = 31**	**NT Group** ***n* = 31**	***p***
Sex, *n* (%)			
Male	27 (87%)	14 (45, 16%)	< 0.001
Female	4 (13%)	17 (54.84%)	
Age, m ± SD	9.61 ± 1.25	9.54 ± 1.28	0.842
EDEI-verbal mental age, m ± SD	7.76 ± 1.02	10.21 ± 1.44	< 0.001
EDEI-mental age, m ± SD	9.75 ± 1.3	9.74 ± 1.48	0.993
Level of study, m ± SD	3.03 ± 1.04	3.48 ± 1.12	0.107

ASD, Autism spectrum disorder; NT, Neurotypical.

M, mean; SD, standard deviation; EDEI, The Differential scales of intellectual efficiency.

The control group (NT children) consisted of 31 verbal children (14 boys and 17 girls) aged between 7 and 12 years (mean age = 9.54 ± 1.28) recruited from different primary schools. Their typical neurodevelopment was verified by a developmental history questionnaire intended for parents. Controls had no history of any psychiatric disorder (assessed using the Mini International Neuropsychiatric Interview for Children and Adolescents (MINI-KID) and had no family history of ASD.

The experimental group (ASD group) consisted of 31 verbal children (4 boys and 27 girls) aged between 7 and 12 years (mean age = 9.61 ± 1.25) recruited from the Department of Child and Adolescent Psychiatry in Razi Hospital (Tunis, Tunisia). The diagnostic of ASD was made by a trained child psychiatrist, according to the fifth edition of the Diagnostic and Statistical Manual of Mental Disorders (DSM-5) criteria and confirmed by the Autism Diagnostic Interview- Revised (ADI-R) (33).

In both groups, Children were excluded from the study if they had sensory deficits, neurological comorbidities, or substance use disorders that could affect the child's cognitive functioning and child's performance during the test. Another inclusion criterion for both groups was a performance IQ >80 (measured using the Tunisian version of the Differential scales of intellectual efficiency (EDEI)-A in its reduced form: the scale I “Vocabulary B” for verbal intelligence and the scale IV “categorical analysis” for non-verbal intelligence (32). The vocabulary scale subtest of the EDEI was used to assess verbal age and only children having a verbal age superior to 6 years were retained in order to select children who could reliably respond to the test questions.

General characteristics of all the studied children are summarized in Table 1.

The study was approved by the Ethics Committee of Razi Hospital. Parents of all participating children were invited to take part in the study and provided written informed consent. The present study adhered to the tenets of the Declaration of Zion et al. (34).

### Measures and procedure

To assess ToM abilities, we used “The Tunisian Social Situations Instrument” (TSSI) (29). The test consists of a downloadable application on any personal computer, tablet, or mobile phone, developed by a research team composed of child psychiatrists and psychologists working in the Child and Adolescent Psychiatry department in Razi Hospital (Tunis, Tunisia). It is a part of a research project titled “social cognition in ASD”, aiming to develop a Tunisian battery for the assessment and remediation of different subdomains of social cognition (verbal and non-verbal ToM, facial emotion recognition, and empathy). The digital design was chosen as it is more attractive for children.

The TSSI is an innovative test, having good reliability and psychometric qualities (Crohnbach alpha = 0.809). The creation of the test were inspired from the Strange Stories of Happé (35), Sally and Anne test (36), and The Faux Pas of Baron-Cohen et al. (17). It is a test of comprehension composed of 9 social situations (stories) with 20 questions: 3 items (questions) for the evaluation of simple comprehension, and 17 questions assessing 4 subdomains of ToM: the attribution of intentions (2 items), epistemic ToM (3 items), affective ToM (3 items), and social clumsiness (9 items). During the validation phase of the test, some items were removed. Therefore, the app has more items assessing social clumsiness. Our test allowed us to study “cognitive ToM” which includes attribution of intentions and epistemic ToM, “affective ToM”, and advanced ToM which is represented by the detection of social clumsiness (or detection of faux pas).

**Figure 1:**
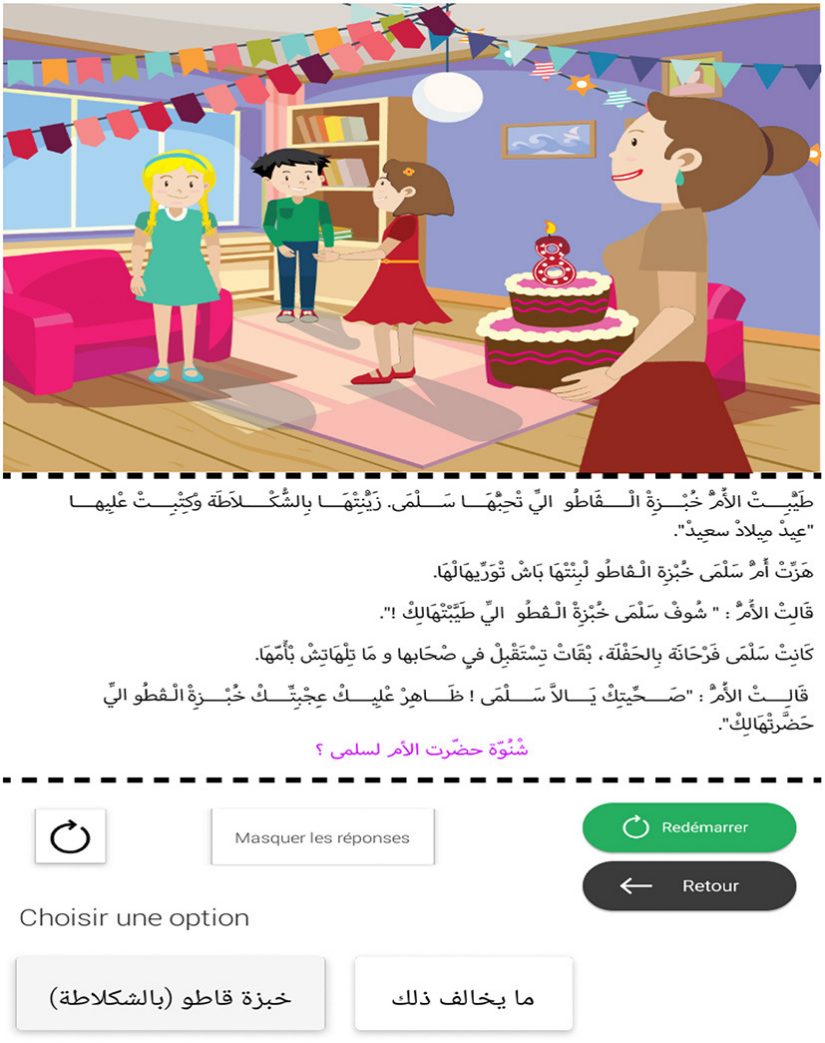
Illustration of the test: “The Tunisian Social Situations Instrument”.

**Figure 2:**
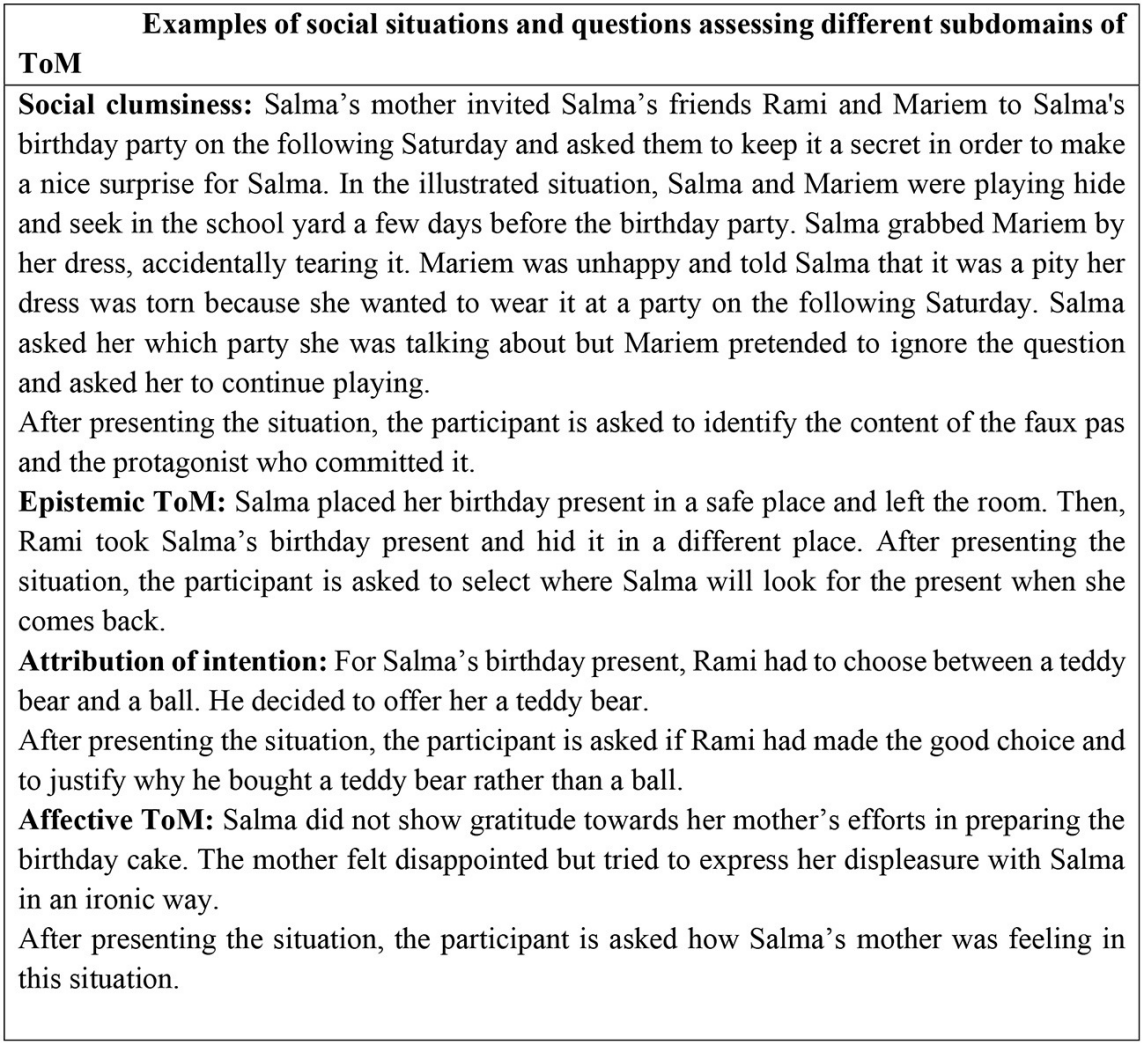
Examples of social situations and questions assessing different subdomains of ToM.

The protagonists of the stories are 3 children (Salma, Meriem, and Rami) and one adult (Salma's mother). The general topic of the stories is Salma's birthday party. Each social situation contains one or various pictures associated with text written in the Tunisian Arabic dialect including questions with simultaneous and automatic reading. Figure 1 illustrates one example of a social situation and Figure 2 shows examples of social situations and questions assessing different subdomains of ToM.

Before starting the test, children were provided with standardized instructions and were asked to select the correct answer after each question. The application generated automatically a total score (out of 20) and a score for each subdomain of ToM without any intervention from the raters. The total duration of the assessment varied between 30 and 50 min.

### Statistical analysis

Statistical analysis was conducted using the Statistical Package for the Social Science (SPSS) version 26 for Windows.

We determined absolute and relative frequencies for categorical variables. We calculated the mean and the standard deviation for continuous variables. In particular, level of studies" was operationalized as a continuous variable and expressed in the number of years of studies that the child has completed.

To compare the sex distribution between groups, we used Pearson's Chi-square. To compare age, mental age, and school grade between the ASD group and the control group, we used Student's *t*-test for independent samples. To compare subscores between matched groups, we used paired-samples *t*-tests.

To examine the association between subscores and sex, we used independent-samples *t*-tests. To examine the associations between the ToM scores and age, mental age, verbal age, and school grade, we used non-parametric Spearman's correlations.

To compare ToM total scores between ASD children and age and mental age-matched NT children, we conducted an analysis of covariance (ANCOVA) with the ToM total scores as dependent variables, the diagnostic group (ASD vs. NT) as a fixed factor, and sex, verbal age, and level of studies as covariates.

To compare ToM subdomain scores between ASD children and age and mental age-matched NT children, we constructed a multivariate analysis of covariance (MANCOVA) with the ToM subdomain scores as dependent variables, the diagnostic group (ASD vs. NT) as a fixed factor, and sex, verbal age, and level of studies as covariates. Effect size was estimated using partial Eta squares (η^2^). Preliminary assumptions for MANCOVA (including normality, linearity, univariate and multivariate outliers, covariance matrices, and multicollinearity) were verified. Pillai's trace test was used because the ToM subdomain scores violated the normality assumption.

A significance level of *p* ≤ 0.05 was used. Holm-Bonferroni's correction of the *p* value was applied in case of multiple comparisons.

## Results

Table 1 summarizes the general characteristics of the ASD group and the NT group. While the mean age and mental age were comparable between groups (groups being matched for these variables), the ASD group had a higher proportion of males and a lower verbal age. The level of study did not differ between the two groups (Table 1).

ToM total score was significantly lower in ASD children compared to matched controls, controlling for gender, verbal age, and level of studies (11.5 ± 2.9 vs. 17.0 ± 2.9 vs., F = 7.783, *p* = 0.007, η^2^= 0.120). In the same analysis, verbal age had a significant strong effect on total ToM score (*p* < 0.001, F = 14.596, η^2^ =.204), whereas gender and level of studies were not found to be associated with ToM total score.

**Figure 3:**
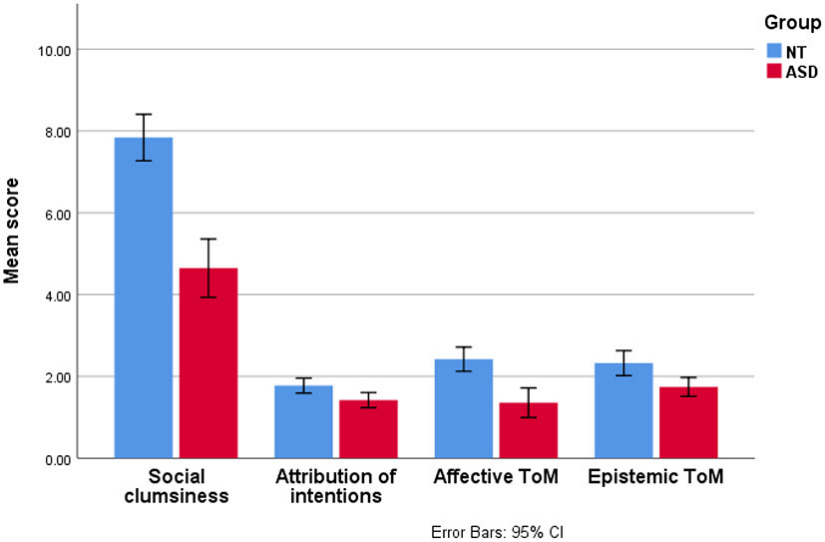
Theory of Mind subdomain scores in children with autism spectrum disorder vs. in neurotypical controls.

Paired-sample *t*-test comparisons showed that all ToM subdomains were significantly lower in children with ASD than in matched controls (adjusted *p* value = 0.019 for attribution of intentions, *p* = 0.014 for epistemic ToM, and *p* < 0.001 for detection of social clumsiness and affective ToM (Figure 3).

In the whole sample, females scored higher in social clumsiness and in affective ToM than males. Their total ToM scores were also significantly higher (Table 2).

**Table 2:** Theory of Mind subdomain scores by sex.

	**Sex**	**N**	**Mean**	**Std. Deviation**	***p* value**
Social clumsiness	Male	41	5.4878	2.21497	< 0.001
	Female	21	7.7143	1.97846	
Attribution of intentions	Male	41	1.5122	0.55326	0.174
	Female	21	1.7619	0.43644	
Affective ToM	Male	41	1.5854	1.09489	< 0.001
	Female	21	2.4762	0.60159	
Epistemic ToM	Male	41	2.0732	0.72077	0.603
	Female	21	1.9524	0.92066	
Total score	Male	41	10.6586	3.91401	0.004
	Female	21	13.9045	3.17130	

ToM, Theory of Mind.

*p* values were adjusted using Holm-Bonferroni's method.

The MANCOVA analysis showed that ToM subscores did not differ significantly between the ASD and the NT group after controlling for sex, verbal age, and level of studies. However, sex and verbal age both had significant strong effects on the ToM subscores (independently of the diagnostic groups) (*p* = 0.025, η^2^ = 0.210; and *p* = 0.006, η^2^ = 0.257, respectively) (Table 3). In particular, out of the four subdomains, sex had a moderate significant effect on epistemic ToM (*p* = 0.005, η^2^ = 0.132, with female sex being associated with higher scores). Verbal age had a moderate to strong effects on all subdomains except attribution of intentions.

**Table 3:** Multivariate analysis of covariance (MANCOVA) comparing ToM subdomain scores in children with autism spectrum disorder to age and mental age-matched controls.

**Effect**	**Pillai's Trace**	**F**	**Sig**.	**Partial Eta Squared**
Gender	0.210	2.825	0.025	0.210
Level of studies	0.085	0.986	0.435	0.085
Verbal age	0.257	3.676	0.006	0.257
Group	0.157	1.972	0.098	0.157

While average ToM subscores (expressed as percentages of the maximum scores for each subdomain) were roughly similar in NT children (between 77.4 and 89.2%, no statistically significant difference), ASD children scored the best in attribution of intentions (71%) (followed by epistemic ToM (58.1%) and the worst in the detection of social clumsiness (51.6%) and affective ToM (45.2%).

## Discussion

The present research aimed to explore the ToM abilities in Tunisian cognitively unimpaired school-aged children with ASD. Our study has several contributions to research in the field of ToM in ASD. First, our research was the first involving an innovative test in a digital format, which makes it more attractive for children with ASD. It has been well established that the use of digital tools attracts the attention of children with ASD to non-verbal social cues because they have difficulty adjusting and switching their attentional focus (37). Second, our research is the first to assess four different subdomains of ToM, including an assessment of three aspects of ToM: cognitive, affective, and advanced ToM (detection of faux pas), by simultaneously presenting social information visually and verbally. Most of published studies have assessed only one subdomain of ToM. Only a few studies have examined different subdomains of ToM, and very few explored advanced ToM specifically in school-aged children (12). Finally, and to our knowledge, our study is the first aiming to examine ToM in Arabic-speaking verbal children using a culturally-validated tool. It is well known that ToM is tightly related to historical, socioeconomic, and cultural factors. Human social and conversational experiences that all societies provide for their young children contributes to the development of psychological understanding (e.g., understanding socially inappropriate behavior) and this is cultivated in a context of social interactions, with a culturally specific developmental trajectory (38–40). Consequently, using culturally sensitive measures for the asssessment of ToM seems crucial.

Our findings have shown that ToM total score was significantly lower in the ASD group than in age and mental age-matched neurotypical controls, after controlling for sex, verbal age, and level of studies, while ToM subscores did not differ significantly between NT children and children with ASD in multivariate analysis. Our study confirmed that ToM is impaired in children with ASD and suggested that ASD children might have more pronounced difficulties in affective ToM and detection of social clumsiness, in comparison to cognitive ToM.

The mixed findings in the literature could be explained by the variation in studied theoretical subdomains of ToM and to age and methodological differences across studies. Several studies using usual tasks to assess ToM in individuals with ASD have shown good performance in this domain, while more complex tests involving social context and studying advanced ToM have shown impairment in ToM (16, 35, 41). Furthermore, some studies that have assessed ToM in adults with ASD (comparing them with a NT sample) have found no difference between the two groups. Authors suggested that adults with ASD may be able to cope with their ToM impairments and correctly answer questions involving ToM (8, 42). In addition, other factors such as high motivation and social attention levels may explain why some individuals with ASD have relatively conserved conceptual understanding of mental states (43).

Recent studies have tried to thoroughly investigate the links between neuropsychological functions and ToM processing in order to better explain the difference in ToM performance across individuals with ASD. Several neuropsychological functions have been associated with ToM performance such as working memory, attention, verbal fluency, verbal flexibility, and executive functioning (21, 22, 26, 44). In the present study, we did not assess these neuropsychological variables. It may be useful to do so in future studies.

In the present study, we found that while NT children performed roughly similarly in all ToM subdomains, the ASD group scored differently across the studied ToM subdomains. The ASD group had the best performance in attribution of intentions (where it did not differ from controls) and the worst performance in detection of social clumsiness followed by affective ToM. Our findings indicate that both affective ToM and detection of social clumsiness or faux pas may remain difficult for children with ASD in comparison to cognitive ToM. Several studies have confirmed this hypothesis. For example, in a recent study of 11, the ASD sample performed significantly worse than their NT peers in affective ToM when they were asked to understand and identify emotions based on desires and beliefs. These results were in line with other studies highlighting difficulties in understanding and reacting appropriately to others' emotions in children with ASD, even in those with the highest cognitive abilities (4, 5, 16, 45–47). Concerning difficulties in detection of faux pas in individuals with ASD, as highlighted in our study, it has been reported that understanding a faux pas means activating two types of mental states: cognitive ToM in order to understand that the person said/did something they should not have, and affective ToM in order to understand that the listener is insulted and hurt. In other words, detecting a faux pas would require someone to, first, detect that the speaker either does not know or does not remember something (unintentional action), and second, to appreciate the emotional impact (usually negative) that the action or comment may have on the listener (22, 48). Thereby, activating simultaneously two types of mental states may remain difficult for individuals with ASD, which explains our findings.

The other focus of our study was to examine the effect of verbal abilities on ToM performance. Our study showed that verbal age had a significant strong effect on ToM total score and moderate to strong effects on all subdomains of ToM except attribution of intentions. Our results are in agreement with many research studies published over the past two decades: 42 hypothesized that individuals with ASD uniquely rely on general reasoning skills and on language abilities for their competence in ToM. In the study by 9, advanced ToM in children and adolescent with ASD was positively associated with participants' verbal abilities. Those finding were, however, not in accordance with those of Bauminger and Kasari (49), showing that children with ASD needed disproportionately advanced linguistic maturity before they could pass standard ToM tasks. Therefore, it seems important to check whether the links between verbal ability and ToM are generalizable to all children with ASD.

In our study, the limited number of female participants in the ASD group did not allow us to operate meaningful statistical analyses of sex performance in ToM and its different subdomains. For this reason, we chose to study gender differences for the entire population and found that female gender was performing better in Total ToM score, in affective ToM and in the detection of social clumsiness. Several studies have shown that females typically perform better in ToM than males both in NT children (50–53) as well as in children with ASD (54).

### Strengths and limitations

Compared with previous research, our study has some strengths. Indeed, we concomitantly assessed multiple subdomains of ToM including cognitive, affective and advanced ToM using an innovative digital tool in which makes it attractive for children with ASD. In addition, we matched the ASD to a neurotypical group for age and mental age to enhance comparability. Nonetheless, several limitations should also be mentioned. Our sample size was mostly imposed by practical difficulties to enroll participants with ASD into the study rather than estimated using an a priori power calculation. In addition, the sex ratio and verbal age were significantly different between the ASD and the control group. This said, we tried to overcome this limitation to some extent by controlling for sex and verbal age in multivariate analyses. Besides this, the limited number of females in the ASD group did not allow us to study gender differences in ASD. Last, the generalizability of our results may be limited by some factors including that the task was only available in digital format and that we excluded non-verbal children and children with sensory deficits.

## Conclusions

In the first study to assess ToM in an Arab population with ASD, we found ASD children to exhibit impairments in ToM, consistently with previous research worldwide. Our findings additionally suggest that affective and advanced ToM, specifically social clumsiness, might be more challenging for ASD children than other ToM subdomains. Future studies with a larger sample size may help us identify which subdomains are the most impaired in order to develop more specific remediation techniques.

## Data availability statement

The original contributions presented in the study are included in the article/supplementary material, further inquiries can be directed to the corresponding author.

## Ethics statement

The studies involving human participants were reviewed and approved by the Ethics Committee of Razi Hospital. Written informed consent to participate in this study was provided by the participants' legal guardian/next of kin.

## Author contributions

SJ: elaboration of the research protocol, assessment of the ASD group, statistical analysis, and redaction of the article. SH: elaboration of Test (Tunisian Social Situations Instrument) and of the research protocol. OR: elaboration of the test and its administration for the control group. ZA, HB, and MH: elaboration of the research protocol. HM: assessment of the ASD group. SO: statistical analysis and correction of the article. AT, SE, and MG: administration of the test for the control group. AN: creation of the test. RF and AM: statistical analysis. AB: elaboration of the test and of the research protocol and correction of the article. All authors contributed to the article and approved the submitted version.

## Conflict of interest

The authors declare that the research was conducted in the absence of any commercial or financial relationships that could be construed as a potential conflict of interest.

## Publisher's note

All claims expressed in this article are solely those of the authors and do not necessarily represent those of their affiliated organizations, or those of the publisher, the editors and the reviewers. Any product that may be evaluated in this article, or claim that may be made by its manufacturer, is not guaranteed or endorsed by the publisher.
